# Repetitive Transcranial Magnetic Stimulation Ameliorates Anxiety-Like Behavior and Impaired Sensorimotor Gating in a Rat Model of Post-Traumatic Stress Disorder

**DOI:** 10.1371/journal.pone.0117189

**Published:** 2015-02-06

**Authors:** Hua-ning Wang, Yuan-han Bai, Yun-chun Chen, Rui-guo Zhang, Huai-hai Wang, Ya-hong Zhang, Jing-li Gan, Zheng-wu Peng, Qing-rong Tan

**Affiliations:** 1 Department of Psychiatry, Xijing Hospital, Fourth Military Medical University, Xi'an, 710032, China; 2 Department of Psychiatry, 91 Hospital of P. L. A., Jiaozuo, 454150, China; University of South Florida, UNITED STATES

## Abstract

**Background:**

Repetitive transcranial magnetic stimulation (rTMS) has been employed for decades as a non-pharmacologic treatment for post-traumatic stress disorder (PTSD). Although a link has been suggested between PTSD and impaired sensorimotor gating (SG), studies assessing the effects of rTMS against PTSD or PTSD with impaired SG are scarce.

**Aim:**

To assess the benefit of rTMS in a rat model of PTSD.

**Methods:**

Using a modified single prolonged stress (SPS&S) rat model of PTSD, behavioral parameters were acquired using open field test (OFT), elevated plus maze test (EPMT), and prepulse inhibition trial (PPI), with or without 7 days of high frequency (10Hz) rTMS treatment of SPS&S rats.

**Results:**

Anxiety-like behavior, impaired SG and increased plasma level of cortisol were observed in SPS&S animals after stress for a prolonged time. Interestingly, rTMS administered immediately after stress prevented those impairment.

**Conclusion:**

Stress-induced anxiety-like behavior, increased plasma level of cortisol and impaired PPI occur after stress and high-frequency rTMS has the potential to ameliorate this behavior, suggesting that high frequency rTMS should be further evaluated for its use as a method for preventing PTSD.

## Introduction

Post-traumatic stress disorder (PTSD) refers to a condition in which exposure to life-threatening trauma results in a characteristic set of features including intrusive memory, where patients persistently experience stress-associated events [1]. The prevalence of a history of post-traumatic stress disorder is estimated at 1 percent in the general population, about 3.5 percent in civilians exposed to physical attack and more than 20 percent in veterans wounded in war [2]. PTSD pathogenesis is still unclear. It is believed that impaired sensorimotor gating (SG) is a contributing factor. SG is a pre-attentive filtering process that allows brain to avoid information overload [3] and properly encode information [4]. Indeed, a number of studies have described a relationship between PTSD and SG dysfunction [5,6]. Although a strong stimulus results in a startle reflex across species a relatively weak sensory stimulus presented 30–500 ms before the strong stimulus will reduce the startle response [7]. This phenomenon, known as the prepulse inhibition (PPI) effect, provides a simple explanation of how dysfunctional SG might contribute to PTSD. By regulating motor and pre-motor systems, PPI reduces responses toward irrelevant information, which is recognized as an operational measurement of SG [8]. Increasing evidence indicates that PPI dysfunction positively correlates with PTSD [9,10,11]. Recently, a clinical study also suggested disrupted sensory filtering in PTSD [12]. However, it remains unclear when precisely impaired SG occurs after stress and how it can be effectively attenuated.

Single prolonged stress (SPS) is a well-established animal model of PTSD [13]which mimics a portion of the neuroendocrine and behavior changes [14] associated with PTSD. For example, in this model the hypothalamus-pituitary-adrenal axis provides rapid negative feedback after administration of glucocorticoid [14] to increase anxiety behavior in the elevated plus maze [15], increase contextual freezing [16], and produces an exaggerated acoustic startle response (ASR) [17]. These changes support the validity of SPS as an animal model of PTSD. Our recent work demonstrated that an inescapable foot electric shock added to a classic SPS model (SPS & electric foot shock, SPS&S) significantly enhances fear responses [18] offering a novel PTSD model. This improved model offers significant changes in open field, elevated plus-maze, and Morris water maze performance reflecting increased anxiety-like behaviors [19] and intrusive memory [20] which are characteristic features of PTSD.

Some pharmaceuticals provide therapeutic benefit for PTSD in humans [21,22] and some of these drugs have been shown to prevent anxiety-like behaviors and cognitive impairments in stressed rats [23]. However, not all patients respond to the currently available pharmacological treatment options for PTSD [24,25]. For example, no significant differences between sertraline (a known PTSD drug) and placebo on any of the efficacy measures at endpoint in a military population [25]. Therefore, non-pharmacologic treatment approaches have been developed. Repetitive transcranial magnetic stimulation (rTMS) is a safe and non-invasive method, and studies have indicated that administration of high-frequency rTMS to the right dorsolateral prefrontal cortex is beneficial for ameliorating PTSD symptoms [26,27]. Although it was primarily utilized for treatment, we hypothesized that rTMS might also provide benefits as an early prevention measure. In this study, we demonstrated that stress-induced anxiety-like behavior and impaired PPI occur after stress and demonstrated that rTMS could attenuate stress-associated behaviors, anxiety-like behaviors and impaired SG, in the SPS&S paradigm.

## Materials and Methods

### 1. Animals

Eighty-eight adult male Sprague-Dawley (SD) rats (nearly 8 weeks old, 180–220 g) were purchased from the Experimental Animals Center of Fourth Military Medical University (Xi’an, China). Four rats were housed per cage in an air-conditioned room (at 22 ± 1°C) with 50–55% relative humidity under a 12-h light/dark cycle and provided with food and water *ad libitum* for 7 days before experimentation. The study protocol was approved by the Committee of Animal Care and Use for Research and Education (CACURE) of the Fourth military Medical University and complied with the National Institutes of Health (NIH) guidelines for the care and use of laboratory animals.

### 2. Study design

**Fig 1:**
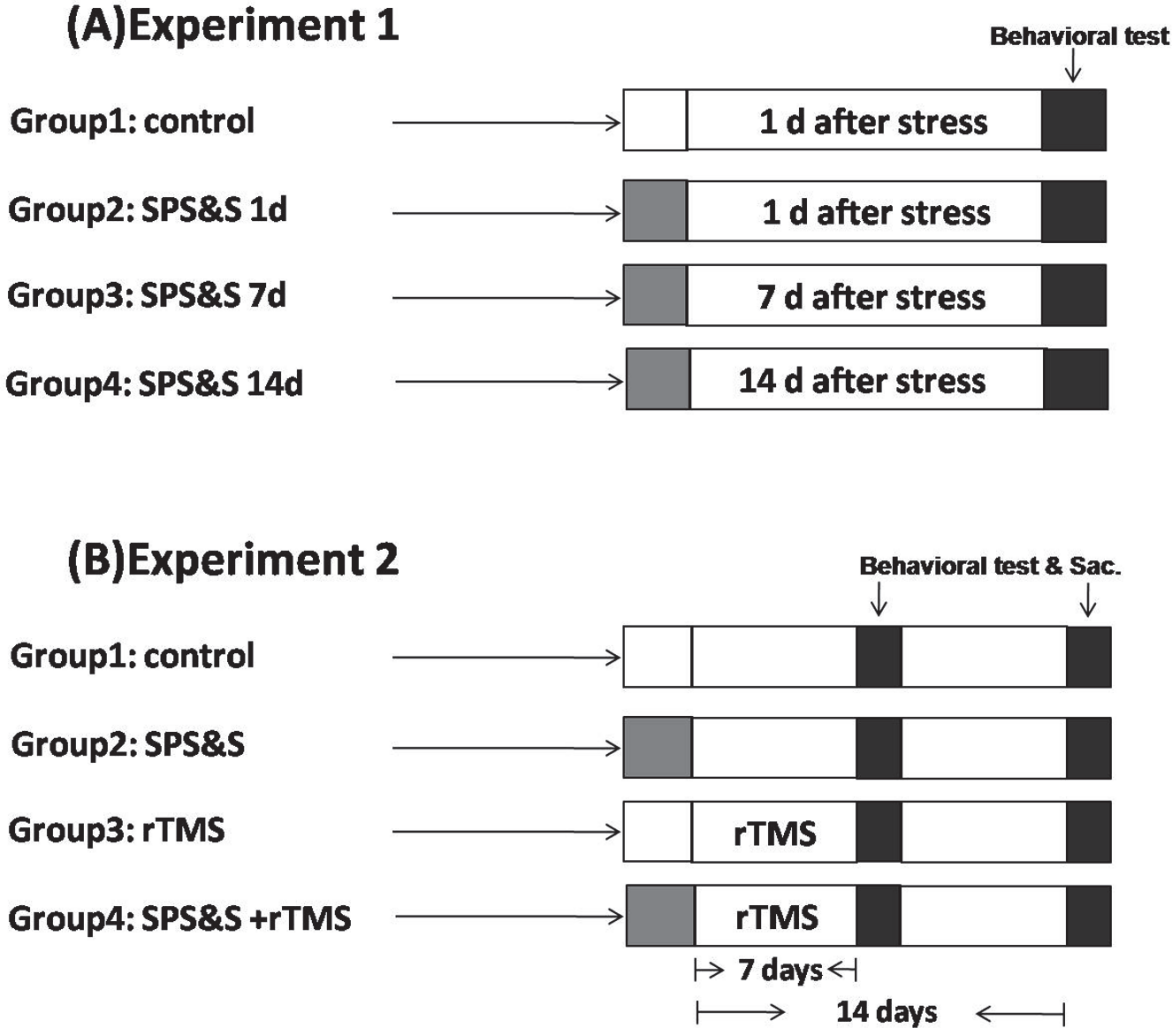
Study design. (A) The animals (no SPS&S) were submitted to a behavioral test 1 day after stress induction in other groups, together with the SPS&S 1 d group. Behavioral test were carried out with SPS&S 7 d and SPS&S 14 d, 7 and 14 days after SPS&S stress induction; (B) After SPS&S and control administered, the rats treated with rTMS for 7 days, then the behavioral tests were given and part of rats were sacrificed for the plasma CORT test and Western blot; The remaining rats were submitted to the behavioral tests and then sacrificed for the plasma CORT test and Western blot 7 days later.

Two experiments were carried out in this study. All animals were allowed one week adaptation before experiment start. In the first experiment, 48 animals were randomly divided into 4 groups (*n* = 12/group) including control, SPS&S 1 d, SPS&S 7 d, and SPS&S 14 d. The rats in control group (no SPS&S) were submitted to a behavioral test 1 day after stress induction in other groups, together with the SPS&S 1d group. Behavioral test were carried out with SPS&S 7 d and SPS&S 14 d, 7 and 14 days after SPS&S stress induction (Fig. 1A).

In the second experiment, 80 rats were randomly divided into 4 groups (n = 20/group): control, SPS&S, rTMS, rTMS + SPS&S. Rats in control group were neither submitted to stress nor treated with rTMS. In the rTMS group, animals were not submitted to stress but treated with rTMS. The SPS&S group included animals submitted to SPS&S stress but without rTMS treatment while the rTMS + SPS&S animals were treated with rTMS after SPS&S stress induction (Fig. 1B). Part of animals (n = 10 for each group) were exposed to behavioral tests at SPS&S 7 d and then they were sacrificed and the expression of c-Fos and glucocorticoid receptor of medial prefrontal cortex (mPFC) were detected by Western blot and the plasma levels of corticosterone (CORT) were detected by Elisa. The remained animals were exposed to behavioral tests at SPS&S 14 d and then they were sacrificed and the expression of c-Fos and glucocorticoid receptor of mPFC were detected, as well as the plasma levels of corticosterone (CORT). All rats were naïve to the experimental apparatus.

### 3. SPS&S animal model

The detailed procedures for the SPS&S procedure were described in previous report [18]. Briefly, rats were restrained for 2 h immediately followed by 20 min forced swimming in 24°C water. A transparent acrylic cylinder (24 cm diameter, 50 cm height) was used for forced swimming, filled to about two-thirds with water. Rat activity during swimming was recorded with an attached CCD camera. After recuperating for 15 min, rats were exposed to diethyl ether until they lost consciousness. Afterwards, the animals were kept in the shock chamber until recovery (about 30 min) and the electric foot shock was applied through the metal grid installed at the bottom of the chamber.

### 4. rTMS treatment

The YRD CCY-1 magnetic stimulation apparatus with a matched circular coil (5-cm inner coil, 6.5-cm outer winding) purchased from Yiruide Co., Ltd. (Wuhan, China) was employed. In accordance with our previous study [28] and another report [29], the rTMS parameters were modified as follows: the stimulating frequency was 10Hz and the stimulating pulse intensity 30% of the rTMS device maximum power (0.7 Tesla). Each train consisted of 51 pulses and was repeated 20 times daily (1020 pulses per day) with an inter-train interval of 15s. These were administered for 7 consecutive days (7140 pulses in total). Rats were administered the magnetic stimulation during inhalation anesthesia (1L/min oxygen with 5% isoflurane for initial anesthesia followed by a reduction to 1–2% isoflurane during the stimulation procedure). Our preliminary data showed that this isoflurane regimen do not affect the assessed behaviors (OFT, EPMT, PPI) in agreement with previous study [30]. Nevertheless, all rats, including control animals, were administered isoflurane to rule out any anesthesia related effects. During stimulation, the center of the coil was placed on the vertex, gently touching the scalp. For sham stimulation of the control group, a powerless coil was placed perpendicular to the scalp.

### 5. Open field test (OFT)

OF was used to measure spontaneous locomotor activity of the animals [31]. The apparatus was composed of four black acrylic plastic boxes (47×47×47 cm) (DigBehav, Jiliang Co., Ltd., Shanghai, China) placed in a soundproof box. Recordings were performed in the soundproof box illuminated by a red light (30 W). Each rat was placed in the center zone at the beginning of testing and horizontal distance traveled was automatically recorded for 10 min by an automatic analyzing system.

### 6. Elevated plus maze test (EPMT)

EPM was used to determine the behavior of approach-avoidance conflict in rats [16]. The apparatus consisted of two opposing arms (50 cm×10 cm) enclosed by 40 cm high side and end walls (closed arms), the other two arms were not installed with walls (open arms) with the entire maze (DigBehav, Jiliang Co. Ltd., Shanghai, China) elevated 50 cm above the floor. During testing, rats were first placed in the central area (10×10 cm) of the maze pointed towards an open arm. Afterwards, activity was recorded for 5 min with an infrared camera in a dark room.

### 7. Prepulse inhibition trial (PPI)

An animal acoustic startle system (Coulbourn Instruments, USA) was used for testing [3]. The apparatus consisted of a sound controller and four chambers. Each chamber consisted of a Plexiglas box resting on a platform which recorded reflex values. Rats were placed in each Plexiglas box and were exposed to four different types of trials after 5 minute acclimation period. The first was a pulse alone trial in which 50 ms of a 108 dB white noise burst without a sound rise/fall was administered. The remaining three were varying prepulse (70 dB, 76 dB, 82 dB) + pulse trials in which 50 ms, 5 KHz sounds were presented before the onset of a 108 dB pulse without a sound rise/fall, with 500 ms between prepulse and pulse. The inter-trial interval ranged from 30 to 45 seconds. The ventilation fans were set to be intermittent and used a minimum/maximum range of 30 to 70%. Each trial was repeated 10 times with a total of 40 trials during approximately 30 minutes. The acoustic startle reflex for each rat was calculated as the average amplitude on trials in which the pulse was presented alone. Percent Prepulse inhibition (PPI) was calculated for each rat using the following equation: %PPI = (1-PP/P) × 100%, where PP represented the average response for prepulse + pulse trial, and P the mean average response with pulse alone.

### 8. CORT measurement by ELISA assay

Blood serums were collected via tail incision centrifuged at 4°Cand the supernatants of brain homogenates from each treatment group were collected and the levels of CORT were measured by a Rat Elisa Kit according to the manufacturer’s instructions (Westang, F3704, China). The sensitivity of the measurements for CORT was 0.15ng/ml.

### 9. Western blotting

Every rat of the normal control and SPS groups was decapitated. The whole brain was quickly removed and placed in an ice-cold dish; the mPFC was dissected according to the atlas by cutting perpendicularly. The mPFC were lysed with SDS-PAGE sample buffer composed of 62.5 mM Tris–HCl, 2% w/v SDS, 10% glycerol, 50 mM DTT, and 0.1% w/v bromphenol blue. After homogenization, whole cell protein was obtained by centrifugation at 12,000g for 10 min and the supernatant was collected. Protein extracts were separated by SDS polyacrylamide gel electrophoresis and then transferred onto PVDF membranes (Millipore). Membrane was blocked with 5% skim milk in Tris buffered saline (TBS) and then incubated overnight at 4°C with mouse anti- Glucocorticoid receptor antibody (1:2000, Abcam, ab2768), rabbit polyclonal to c-Fos (1:2500, Abcam, ab190289) and mouse anti-β-actin antibody. After washing, membranes were incubated with a horseradish peroxidase (HRP)-conjugated IgG sencondary antibody in accordance with the origin of the primary antibody for 1 h at room temperature. After wash, the antibody-reactive bands were visualized on X-ray film using super ECL plus detection reagent (Supersignal west pico chemiluminescent substrate, Thermo, USA, 34077).

### 10. Statistical analysis

SPSS 16.0 for Windows (SPSS Inc., Chicago, IL, USA) was used to analyze the data, expressed as means ± standard error of the mean (SEM). OF, EPM and PPI indices were analyzed using one-way or two-way analysis of variance (ANOVA) across groups. PPI data among sound pressure levels in the same group were analyzed using repeated measures ANOVA. The least significant difference (LSD)-*t* method was further used as a post hoc test to detect group differences with homogeneity of variance, or Dunnett T3 was used. All tests were two-sided and statistical significance was defined as *P* < 0.05.

## Results

### 1. Spontaneous locomotor activity in a rat model of PTSD

**Fig 2:**
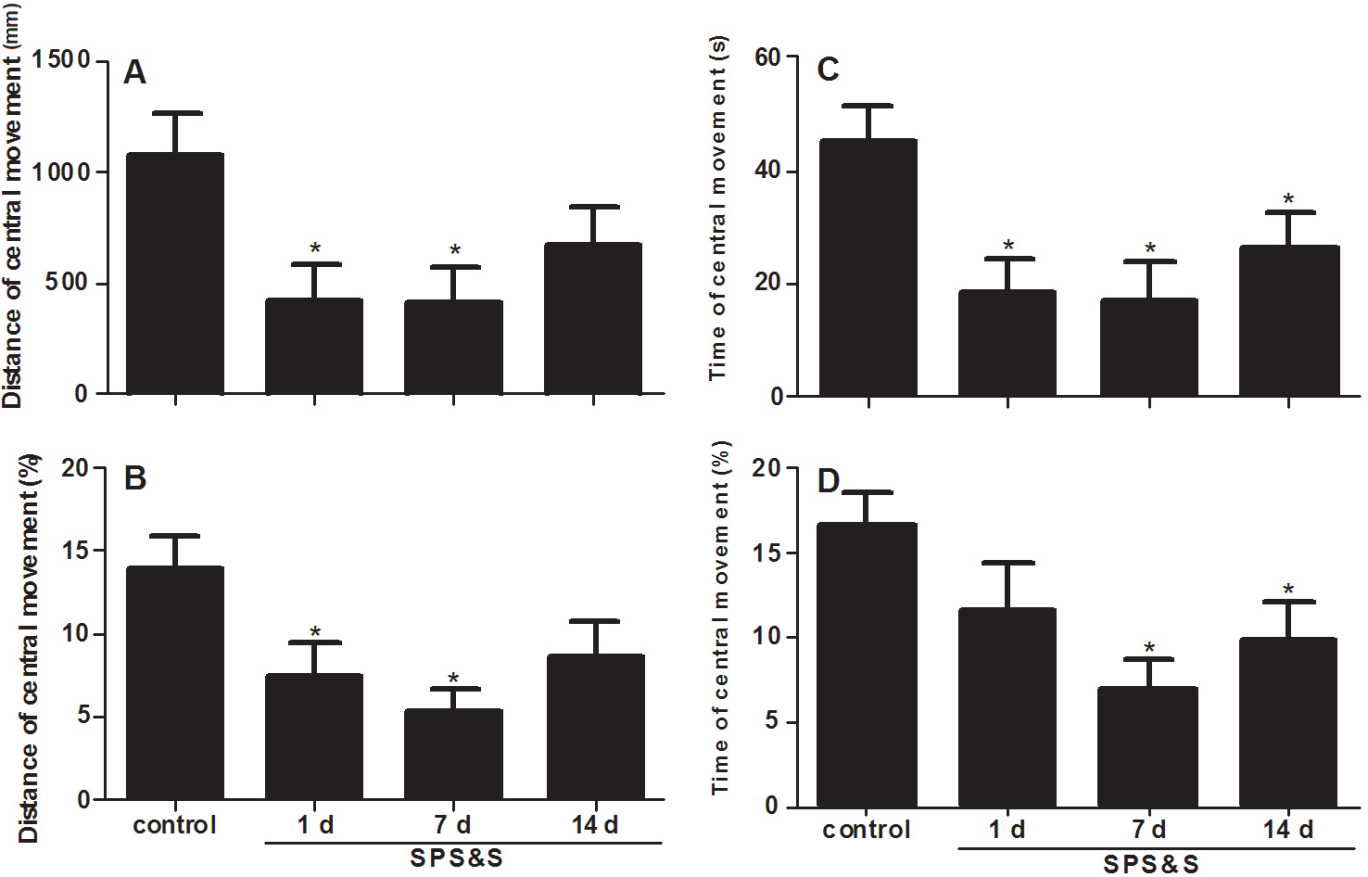
Spontaneous locomotor activity in a rat model of PTSD. Total distance of movement (A), time of central movement (B), distance and time of central movement relative to overall levels (%) values (C, D). Data are expressed as mean ± standard error of the mean (SEM) (*n* = 12 per group). *P < 0.05 *vs*. control.

As shown in Fig. 2, there was no statistically significant difference among the four groups for the total distance of movement (*F*3, 44 = 2.620, *P* = 0.063) and the total time of movement *F*3, 44 = 1.046, *P* = 0.384). However, significant differences were found among the four groups in distance of central movement (*F*3, 44 = 3.363, *P* = 0.027), time of central movement (*F*3, 44 = 4.015, *P* = 0.013), percent distance of central movement (*F*3, 44 = 3.686, *P* = 0.019) and percent time of central movement (*F*3, 44 = 3.325, *P* = 0.031). Compared to the control group, the SPS&S 1 day group had a significantly reduced distance of central movement, less time of central movement and less percent distance of central movement (all *P* < 0.05). On the other hand, the SPS&S 7 day group demonstrated a reduction (all *P* < 0.05) of all four indices, distance of central movement, percent distance of central movement, time of central movement, and percent time of central movement. However, only time of central movement and percent time of central movement were reduced in the SPS&S 14 day group (both *P* < 0.05).

### 2. Behavior of approach-avoidance conflict in a rat model of PTSD

**Fig 3:**
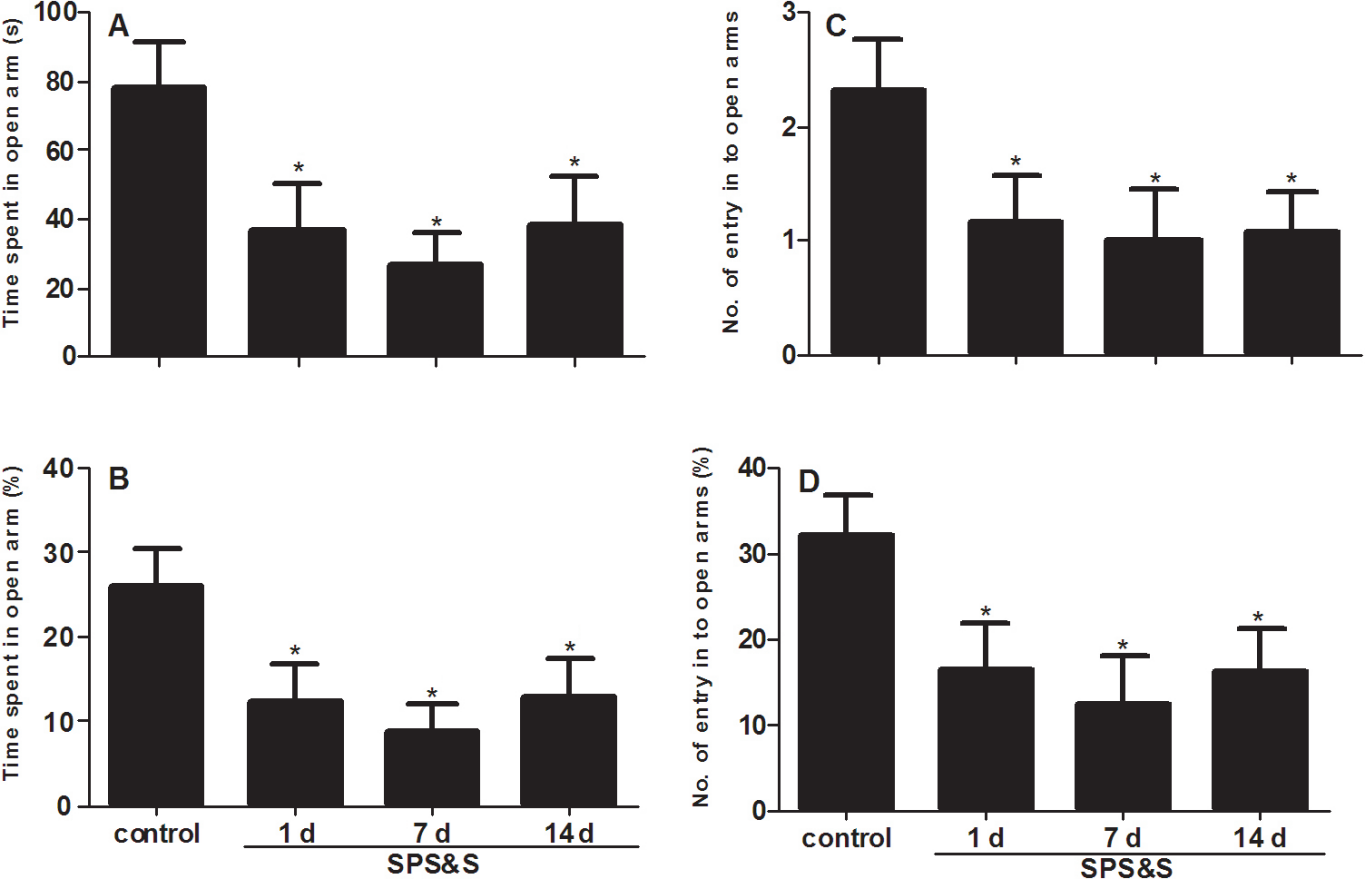
Behavior of approach-avoidance conflict in a rat model of PTSD. Time spent in open arms (A), number of entries into open arms (B) and their relative to overall levels (%) values (C, D) tested in the elevated plus maze. Data are expressed as mean ± SEM (*n* = 12 per group). *P < 0.05 *vs*. control.

As shown in Fig. 3, significant differences were found among the four groups in time spent in open arms (*F*3, 44 = 3.204, *P* = 0.032), with 77.93 ± 13.44, 36.62 ± 13.42, 26.43 ± 9.59, and 46.33 ± 14.66 s obtained for controls, and rats evaluated at 1, 7, and 14 days after SPS&S, respectively. Similar results were obtained for percent time spent in open arms, with 25.98 ± 4.48, 12.21 ± 4.47, 8.81 ± 3.2, and 15.44 ± 4.89% (*F*3, 44 = 3.204, *P* = 0.032) and percent numbers of entry into the open arms, with 32.19 ± 4.63, 16.51 ± 5.23, 12.55 ± 5.46, and 16.38 ± 4.88% (*F*3, 44 = 2.968, *P* = 0.042) for control animals and rats evaluated at 1, 7, and 14 days after SPS&S, respectively. However no significant difference was obtained for absolute numbers of entry into the open arms (*F*3, 44 = 2.392, *P* = 0.081). These data indicated that rats submitted to SPS&S and evaluated 1 and 7 and 14 days showed a reduction in time spent in the open arms and percent time spent in the open arms compared to the control group (all *P* < 0.05), although the SPS&S 14 day group showed a slight increase compared with other SPS&S 7 animals. Each SPS&S group also showed a reduction in percent number of entries into the open arms (all *P* < 0.05).

### 3. Sensorimotor gating in a rat model of PTSD

**Fig 4:**
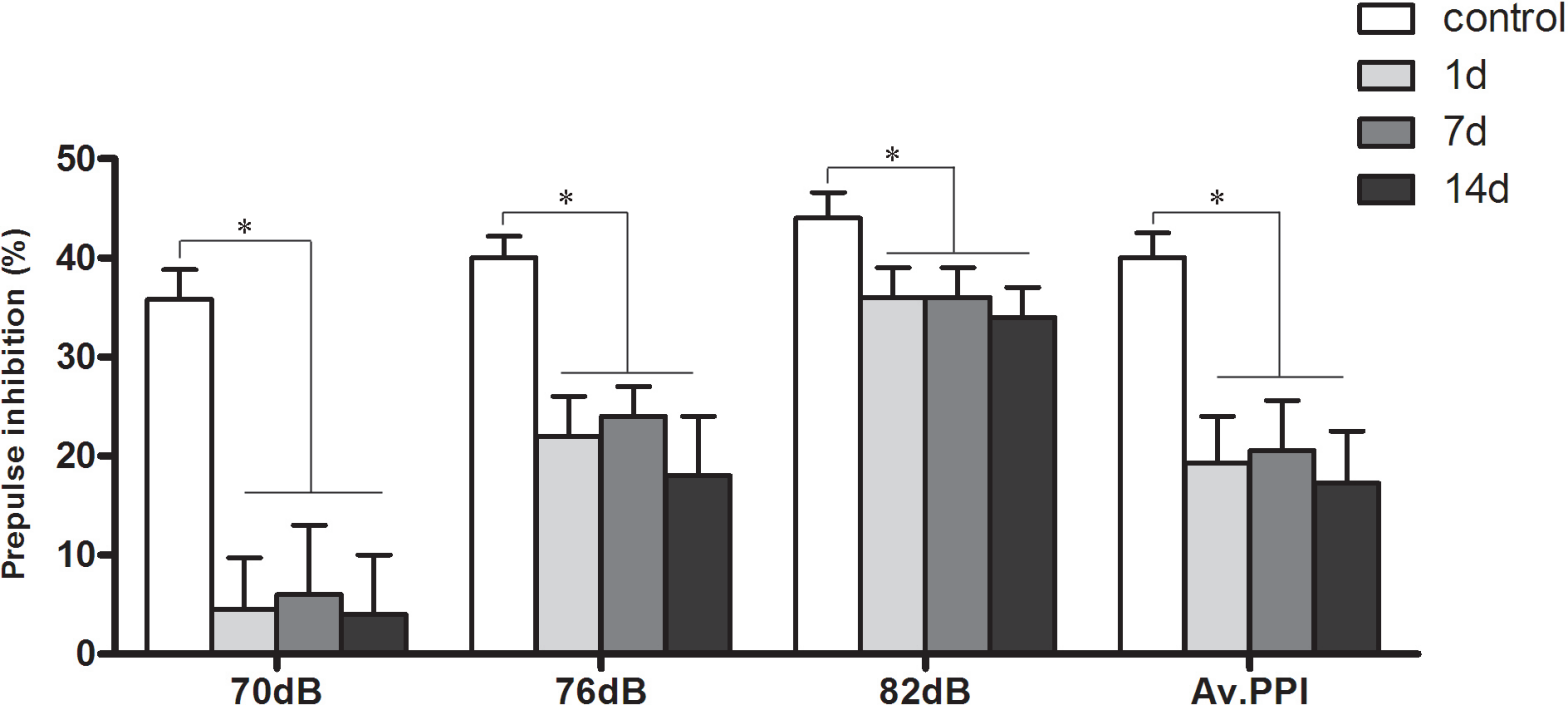
Sensorimotor gating in a rat model of PTSD. %PPI (Prepulse inhibition (%)) among four groups (*n* = 12 per group). Mean ± SEM of %PPI by prepulses with different intensities (72, 76, and 82 dB) and calculated all over the three prepulse intensities in each group (Av.PPI is average of total 70–82 dB %PPI in each group).*P < 0.05.

A statistically significant difference was observed in average %PPI (70–82 dB) among the four groups (*F*3, 44 = 2.869, *P* = 0.047) as shown in Fig. 4. Indeed, average %PPI (70–82 dB) of control animals (40.03 ± 2.50%) was higher than those found in the three SPS&S groups (all *P* < 0.05), including rats evaluated at 1 (19.29 ± 4.72%), 7 (20.58 ± 5.01%), and 14 (17.26 ± 5.24%) days.

### 4. Effect of rTMS on spontaneous locomotor activity in a rat model of PTSD

**Fig 5:**
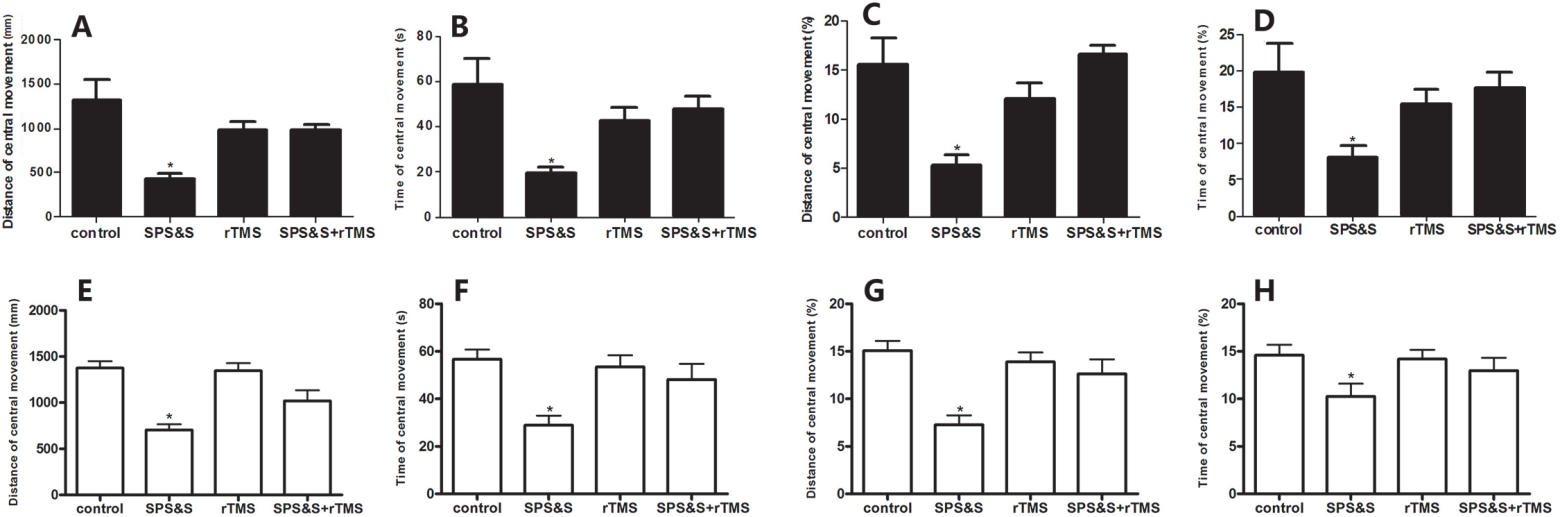
Effect of rTMS on spontaneous locomotor activity in a rat model of PTSD. Distance of central movement and Time of central movement in absolute after SPS&S treatment for 7 day (A, B) or 14 day (E, F) and relative to overall levels (%) values after SPS&S treatment for 7 day (C, D) or 14 day (G, H) tested in open field. Data are expressed as mean ± SEM (*n* = 10 per group). *P< 0.05 *vs*. control or SPS&S + rTMS group.

Significant differences were observed among the four groups in distance of central movement (*F*3, 36 = 7.360, *P* = 0.001) (Fig. 5A), time of central movement (*F*3, 36 = 5.319, *P* = 0.004) (Fig. 5B), percent distance of central movement (*F*3, 36 = 6.705, *P* = 0.001) (Fig. 5C) and percent time of central movement (*F*3, 44 = 3.707, *P* = 0.020) (Fig. 5D) at SPS&S 7 d. In addition, compared with the SPS&S group, rats treated with rTMS + SPS&S significantly increased the distance of central movement (978.03 ± 73.91 *vs*. 421.16 ± 65.73), percent distance of central movement (11.67 ± 0.88 *vs*.5.40 ± 0.93), time of central movement (47.93 ± 5.28 *vs*. 19.40 ± 2.81), and percent time of central movement (17.60 ± 2.26 *vs*. 8.14 ± 1.55) (all *P* < 0.05).

There were also significant differences among the four groups in distance of central movement (*F*3, 36 = 12.169, *P* < 0.01) (Fig. 5E), time of central movement (*F*3, 36 = 4.892, *P* = 0.006) (Fig. 5F), percent distance of central movement (*F*3, 36 = 6.583, *P* = 0.001) (Fig. 5G) and percent time of central movement (*F*3, 44 = 3.269, *P* = 0.334) (Fig. 5H) at SPS&S 14 d. In addition, compared with the SPS&S group, rats treated with rTMS + SPS&S significantly increased the distance of central movement (1027 ± 191.28 *vs*.693.31 ± 133.68), percent distance of central movement (12.68 ± 3.74 *vs*.6.22 ± 3.62), time of central movement (47.86 ± 25.17 *vs*. 28.69 ± 14.63), and percent time of central movement (14.08 ± 3.08 *vs*.9.81 ± 2.74) (all *P* < 0.05).

### 5. Effect of rTMS on behavior of approach-avoidance conflict in a rat model of PTSD

**Fig 6:**
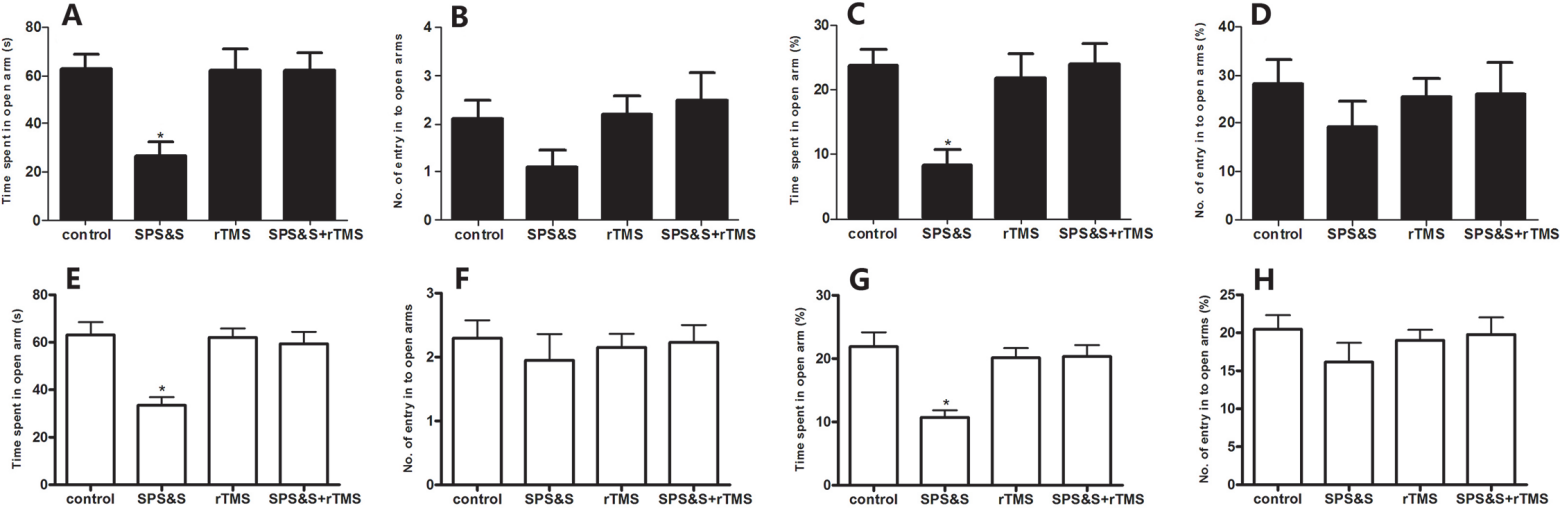
Effect of rTMS on behavior of approach-avoidance conflict in a rat model of PTSD. Time spent in open arms and number of entries into open arms in absolute after SPS&S treatment for 7 day (A, B) or 14 day (E, F) and relative to overall levels (%) values after SPS&S treatment for 7 day (C, D) or 14 day (G, H) tested in the elevated plus maze. Data are expressed as mean ± SEM (*n* = 10 per group). *P< 0.05 *vs*. control or SPS&S + rTMS group.

At SPS&S 7 d, significant differences were found among the four groups in time spent in open arms (*F*3, 36 = 6.368, *P* = 0.001) and percent time spent in open arms (*F*3, 36 = 6.441, *P* = 0.001) (Fig. 6A and 6C). However, no significant difference was obtained for percent and number of entries into open arms (*F*3, 36 = 0.544, *P* = 0.656, *F*3, 36 = 2.017, *P* = 0.129) as shown in Fig. 6B and 6D. Here also, compared with the SPS&S group, rats treated with rTMS + SPS&S significantly increased the time spent in open arms (62.15 ± 7.01 *vs*. 26.84 ± 5.28), and percent time spent in open arms(24.01 ± 0.03 *vs*. 8.42 ± 2.27) (both *P* < 0.05).

There were also significant differences among the four groups in time spent in open arms (*F*3, 36 = 18.221, *P* < 0.01) and percent time spent in open arms (*F*3, 36 = 8.207, *P* < 0.01) (Fig. 6E and 6G). However, no significant difference was obtained for percent and number of entries into open arms (*F*3, 36 = 0.081, *P* = 0.970, *F*3, 36 = 0.860, *P* = 0.469) as shown in Fig. 6F and 6H at SPS&S 14 d. Here also, compared with the SPS&S group, rats treated with rTMS + SPS&S significantly increased the time spent in open arms (54.35 ± 9.61 *vs*. 30.98 ± 8.01), and percent time spent in open arms(20.09 ± 4.59 *vs*.10.39 ± 4.02) (both *P* < 0.05).

### 6. Effect of rTMS on sensorimotor gating in a rat model of PTSD

**Fig 7:**
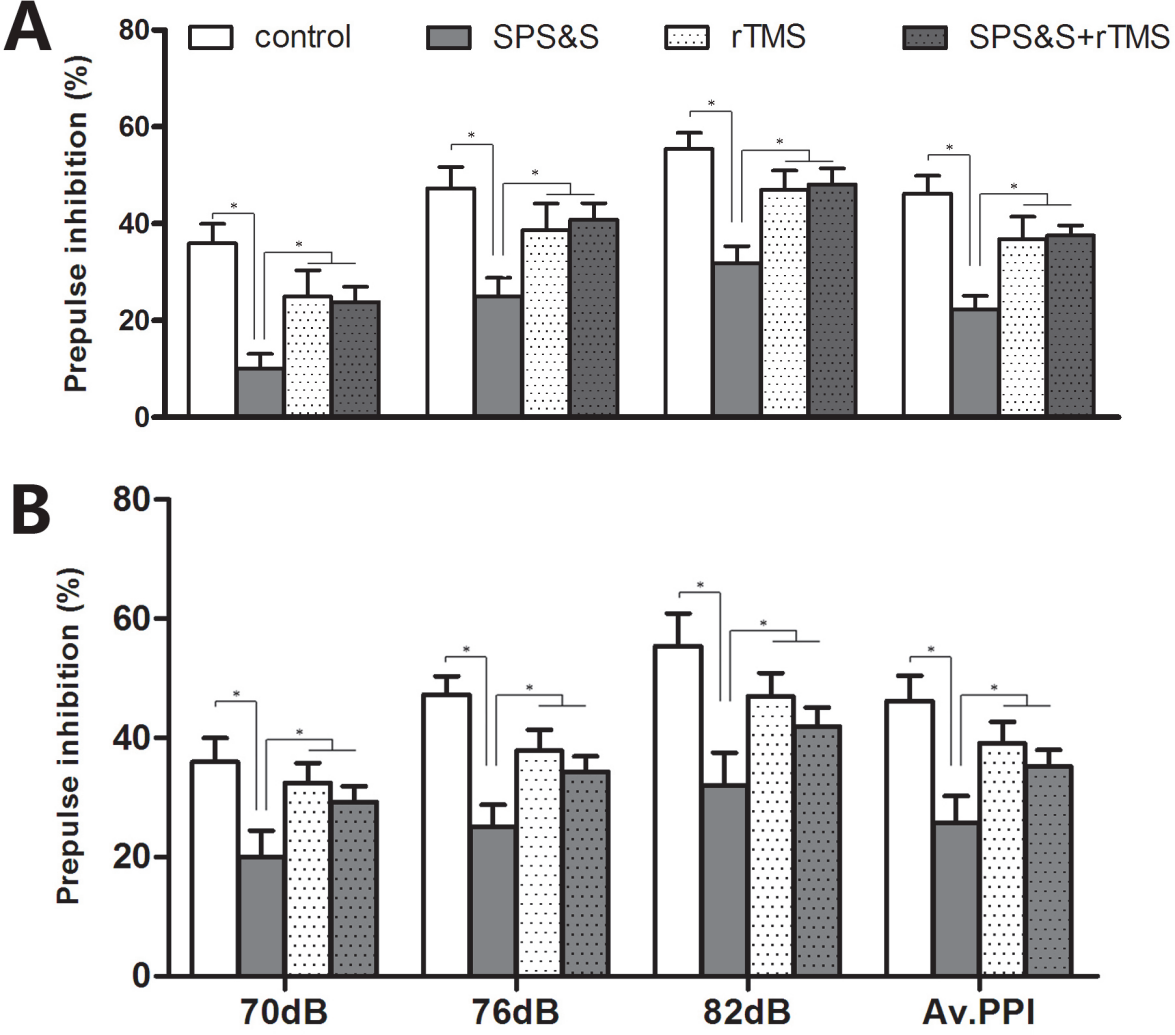
Effect of rTMS on sensorimotor gating in a rat model of PTSD. %PPI among four groups after SPS&S treatment for 7 day (A) or 14 day (B). Mean ± SEM of %PPI by prepulses with different intensities (72, 76, and 82 dB) and calculated over all three prepulse intensities in each group (Av. PPI is the average of total 70–82dB %PPI in each group). *P < 0.05.

We observed a statistically significant difference in average %PPI (70–82dB) between the SPS&S group (22.30 ± 2.82) and the remaining three groups at SPS&S 7 d, including control animals (46.25 ± 3.63) and rats treated with rTMS (37.53 ± 4.27) and SPS&S + rTMS (37.58 ± 2.08) (*F*3, 36 = 8.456, *P* < 0.001) as shown in Fig. 7A. Furthermore, %PPI values obtained for the SPS&S group were significantly lower compared with the other groups at all intensities (all *P* < 0.05).

There were also statistically significant differences in average %PPI (70–82dB) between the SPS&S group and the remaining three groups at SPS&S 14d (*F*3, 36 = 14.35, *P* < 0.01) as shown in Fig. 7B. Furthermore, %PPI values obtained for the SPS&S group were also significantly lower than that of the other groups at all intensities (all *P* < 0.05).

### 7. Effect of rTMS on the expression of c-fos and glucocorticoid receptor (GR) in the mPFC and the plasma level of cortisol

**Fig 8:**
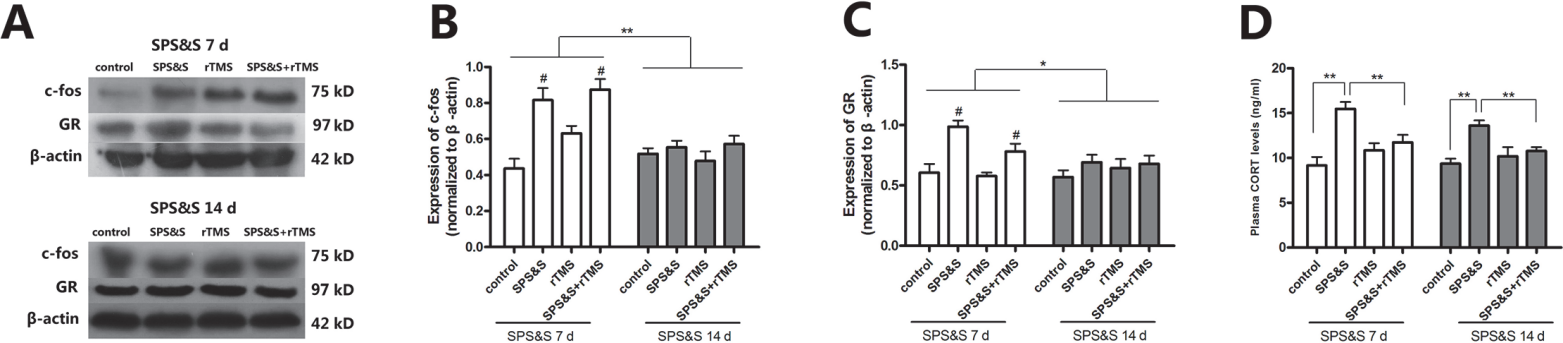
Effect of rTMS on the expression of c-fos and glucocorticoid receptor (GR) in the mPFC and the plasma level of cortisol. Representative bands (A) and the densitometric analysis (B, C) for the protein expression of c-fos and GR and the plasma level of CORT (D) at SPS&S 7 d and SPS&S 14 d. **P < 0.01, *P < 0.05, #*P* < 0.01 *vs*. control or rTMS group, ^▲^ *P* < 0.05 *vs*. control.

At SPS&S 7 d, significant differences were observed among the four groups in the expression of c-fos (*F*3, 17 = 12.058, *P* < 0.01) (Fig. 8B) and GR (*F*3, 17 = 11.370, *P* < 0.01) (Fig. 8C) in the mPFC. Meanwhile, compared with the control or rTMS group, rats treated with SPS&S or rTMS + SPS&S significantly increased the expression of c-fos and GR (all *P* < 0.01); There was no significant difference among the four groups in the expression of c-fos (*F*3, 20 = 0.740, *P* = 0.540) (Fig. 8B) and GR (*F*3, 17 = 0.969, *P* = 0.432) (Fig. 8C) in the mPFC at SPS&S 14 d. Two-way ANOVA further revealed significant differences of stress factor (control *vs*. SPS&S *vs*. rTMS *vs*. rTMS + SPS&S) in the expression of c-fos (*F* = 10.384, *P* < 0.01) and GR (*F* = 7.202, *P* = 0.001), there were also significant differences of time factor (SPS&S 7 d *vs*. SPS&S 14 d) in the expression of c-fos (*F* = 19.881, *P* < 0.01) and GR (*F* = 4.372, *P* = 0.043) (Fig. 8D).

There were also significant differences among the four groups in the plasma level of cortisol at SPS&S 7 d (*F*3, 32 = 10.507, *P* < 0.01) and SPS&S 14 d (*F*3, 36 = 10.087, *P* < 0.01). In addition, compared with the SPS&S group, rats in control or rTMS + SPS&S group showed significantly lower cortisol level at both SPS&S 7 d and SPS&S 14 d. (all *P* < 0.01).

## Discussion

Several studies have suggested that decreased central movements in the OFT [32] and decreased open-arm parameters in the EPMT paradigm represent surrogates for anxiety-like behavior [33]. Our data described here suggest that SPS&S is an effective model to mimic stress-related anxiety behavior as a component of the PTSD characteristics. Indeed, we found that stress reduced central movements in the OFT and open-arm movements in the EPMT without causing over all locomotor impairment, indicating that the stressed rats exhibited persistent anxiety-like behaviors. These findings further validated the SPS&S model for PTSD. Impairment of PPI represents SG dysfunction which could be explained by the theory of attentive processing. PPI refers to a situation in which the subject notices the weaker or secondary stimulus thus transferring attention from the stronger stimulus, and results in a reduced startle response [34]. Previous studies using human subjects have demonstrated that selectively paying attention to the prepulse stimulus enhances the magnitude of PPI [34,35], indicating that PPI is a reflection of general attention processing ability and therefore relevant to provide protection for the pre-attention stimulus [36]. The failure of patients with PTSD to inhibit negative emotion and memories of traumatic experiences may imply a defect in PPI. They may suffer impairment of intra-attention and be unable to restrain the onset or recall of negative information, suggesting that PPI dysfunction is a component of the pathophysiologic mechanism of PTSD [9,10,11,12]. This idea is further supported by animal studies. For example, stressed alpha-2C adrenergic receptor knockout mice have impaired PPI [37]. In addition, a study comparing predator stress versus footshock stress in rats demonstrated that changes in c-fos expression in different regions correlated with effects on PPI [38]. In the present work, we provide evidence for the first time that impaired PPI and anxiety behavior occur in SPS&S rats with a reduction in PPI arising immediately and persisting for some time: traumatic events can induce impairment immediately after the stress and early intervention is therefore needed after the traumatic exposure.

Two approaches have been proposed as early interventions for PTSD. In the first, action precedes the stress with the intention of enhancing resistance to stress while in the second approach, treatment is introduced after in order to prevent establishment of a stress related syndrome [27]. Since many traumatic incidents are unpredictable, the latter approach is likely the one that is feasible. Accordingly, we determined whether rTMS, a well-established therapeutic method PTSD treatment, offered benefits as early intervention after stress. Prior studies have indicated that administration of high-frequency rTMS to the right dorsolateral prefrontal cortex is beneficial for ameliorating PTSD symptoms [26,27]. Our data presented herein demonstrated for the first time that chronic administration of rTMS has anxiolytic effect on stress-related anxiety-like behaviors without affecting control rats.

Several studies have suggested that DAergic and 5-HTergic systems are associated with anti-PTSD treatments [39,40]. Kanno et al. [41] found that chronic rTMS, but not acute rTMS improves behavior of rats on the plus-maze, which might involve the serotonergic system. In addition, there are several other biological aspects that should be considered to understand the mechanism of rTMS, including various aspects of stress biology, immune function disruptions, neural structure, and function, as well as circadian rhythms [42]. Some animal studies also suggest that rTMS affects neurotransmitter systems of glutamate and gamma-aminobutyric acid (GABA); stress-induced activity of the hypothalamic-pituitary-adrenal (HPA) system; and neurotrophic signaling factors, such as brain derived neurotrophic factor (BDNF) [43,44,45]. High-frequency rTMS has been reported to regulate brain activity and increase cortical excitability [46]. In addition, neural activity and corresponding hemodynamic responses induced by rTMS have been shown in the cat visual cortex [47]. Our previously studies showed that high frequency (15 Hz) rTMS could elevate the expression of BDNF in the hippocampus and decrease the level of plasma ACTH and CORT in chronic unpredictable mild stress (CUMS) rats [28,48]. In the present study, we found the CORT level was increased after SPS&S which was in accordance with previously study [49], and high frequency rTMS treatment also alleviated this situation, the underlying mechanisms still need further studied.

It is well known that the main pathway of PPI is cortico-striato-pallido-thalamic (CSPT) [50] the inferior colliculus [51], superior colliculus [52] and pallidotegmental area [53] playing key roles. The medial prefrontal cortex (mPFC) is a higher order structure that controls stress and fear responses of the hippocampus and amygdale [54], and it plays an important role in the regulation of fear, anxiety and aggression [55]. In particularly, PPI is normally regulated by the mPFC [56,57], and studies have showed that mPFC is closely involved in the pathogenesis of PTSD [58,59]. We speculate that stress affects mPFC or other brain regions that are sensitive to magnetic stimulation at early stages after stress and rTMS stimulates those circuits to protect against PPI impairments. In the present study, we found that chronic rTMS could prevent PPI impairment with little effect on control rats; the expression of c-fos and GR in the mPFC was increased 7 d after SPS&S, and was recovered 14 d after SPS&S, and chronic rTMS also elevated the expression of c-fos but did not affect the expression of GR in the mPFC. It is well known that the expression of c-fos and GR was increased in the animal model of PTSD [60,61], and the expression of c-fos and GR was changed immediately after stress, it is hard to speculate the involvement of c-fos and GR expression of mPFC on the effect of rTMS. Further studies are required to optimize the conditions of rTMS for maximizing its preventative potential.

In summary, stress rapidly causes stress-induced anxiety-like behavior and altered PPI, which could be prevented by high-frequency rTMS. Our findings suggest that high frequency rTMS should be further evaluated for its use as a method for preventing PTSD.
